# Characterization of the complete chloroplast genome of Sanming wild banana (*Musa itinerans*) and phylogenetic relationships

**DOI:** 10.1080/23802359.2019.1642167

**Published:** 2019-07-17

**Authors:** Yong-Yan Zhang, Fan Liu, Na Tian, Jing-Ru Che, Xue-Li Sun, Zhong-Xiong Lai, Chun-Zhen Cheng

**Affiliations:** College of Horticulture, Fujian Agriculture and Forestry University, Fuzhou, P. R. China

**Keywords:** *Musa itinerans*, wild banana, chloroplast genome, phylogenetic relationship analysis

## Abstract

Sanming wild banana (*Musa itinerans*) is a cold resistant wild banana found in Fujian province of China. In this study, we characterized its complete chloroplast genome using BGISEQ-500 sequencing. The chloroplast genome is 171,815 bp in size, containing a pair of IR regions (35,142 bp), a large single copy region (89,995 bp), and a small single copy region (11,464 bp). The whole chloroplast genome contains 111 unique genes, including 78 protein-coding genes, 29 tRNAs, and 4 rRNAs. Phylogenetic maximum likelihood analysis revealed that Sanming wild banana showed the closest relationship with *Musa itinerans* that collected from Yunnan Province of China.

China is rich of wild banana species (Li et al. 2017), many of which showed high resistance to both abiotic and biotic stresses, such as cold and banana wilt disease (Li et al. 2015). In our previous study, we identified that Sanming wild banana showed the highest cold resistance (Liu, Cheng, Chen, et al. 2018; Liu, Cheng, Lin, et al. 2018), suggesting that it has great potential to be used in banana cold resistance breeding. Based on its phenotype characteristics, it was categorized into *Musa itinerans*, which is native to Southeast Asia and subtropical China (Häkkinen et al. 2008). However, molecular evidence that can clarify its evolution position and relationship with other banana species is limited. In this study, we assembled and characterized its complete chloroplast genome using BGISEQ-500 (BGI, Shenzhen, China) sequencing data and explored the phylogenetic relationship with other plant species, which can be used for the revealing of its evolution position and can also be subsequently used for the cold resistance genetic breeding and the evolutionary study of banana.

The specimen of Sanming wild banana was isolated from the banana germplasm resources nursery of Institute of Horticultural Biotechnology, Fujian Agriculture and Forestry University in Fuzhou (Fujian, China, 26°04'37.00''N;119°14'33.47''E) and the DNA stored at the Fujian Agriculture and Forestry University (No. FAFUYSJ01). High-quality genomic DNA was used for BGISEQ-500 sequencing (BIG, Shenzhen, China). Approximately 1.2 Gbp high-quality reads were obtained, and clean reads were aligned to chloroplast genomes of *Musa acuminate* (Genbank accession No. HF677508.1), *Musa balnisiana* (Genbank accession No. NC_028439.1) and *Musa itinerans* (Genbank accession No. NC_035723.1) and then further assembled into contigs using CLC Genomics Workbench v8.0 (CLC Bio, Aarhus, Denmark). The assembled chloroplast genome was assembled annotated using DOGMA (Wyman et al. 2004) and annotation correction was performed using Geneious (Kearse et al. 2012). The annotated chloroplast genome has been deposited in Genbank with the accession number MN087226.

The complete chloroplast genome of Sanming wild banana is 171,815 bp in size, containing a pair of inverted repeat (IR) regions of 35,142 bp, a large single copy region of 89,995 bp, and a small single copy region of 11,464 bp. The chloroplast contains 111 unique genes, including 78 protein-coding genes, 29 tRNA genes, and 4 rRNA genes. Most of them (89) occur as a single copy, but 10 protein-coding genes (i.e. *ycf2*, *ycf1*, *rps19*, *rps15*, *rps12*, *rps7*, *rpl23*, *rpl2*, *ndhH*, and *ndhB*), 8 tRNA genes (i.e. *trnV-GAC*, *trnR-ACG*, *trnN-GUU*, *trnL-CAA*, *trnI-GAU*, *trnI-CAU*, *trnH-GUG*, and *trnA-UGC*) and all the 4 rRNA genes (*rrn4.5*,* rrn5*,* rrn16 *and* rrn23*rRNA) occur in double copies. The overall nucleotide composition of the chloroplast genome is: 31.5% A, 32.0% T, 18.5% C, and 18.0% G, with the total GC content of 36.5%.

For phylogenetic maximum-likelihood analysis, we downloaded the chloroplast genomes of 16 plant species belonging to Scitamineae from GenBank to access the relationship of Sanming wild banana with them. Sequence alignment was then performed using HomBlocks pipeline (Bi et al. 2018). RAxML-HPC2 on XSEDE version 8.2.10 (Stamatakis 2014) was used to construct a maximum-likelihood tree, for which 1000 bootstrap replicates were used to calculate the bootstrap values. Result showed that Sanming wild banana showed a closest relationship with *Musa itinerans*, followed by *Musa balnisiana* and *Musa textills* (Figure 1), all from the family Musaceae, indicating that Sanming wild banana belongs to *Musa itinerans* of Musaceae. There are many wild banana species distributed in China, and the relationships among them and with other banana species need to be clarified. Our present study revealed that the chloroplast genome size of Sanming wild banana reported in this study is larger than that *Musa itinerans* collected in Yunnan Province, China (Li et al. 2017), and their differences mainly occurred in the intergenic sequences. Thus, it was suspected that the intergenic sequences of the chloroplast can be used in the phylogenetic analysis of wild banana species. Moreover, the complete chloroplast of Sanming wild banana would be subsequently used in the evolutionary studies of Musa species and would provide genetic information for the banana cold resistance breeding.

**Figure 1.**
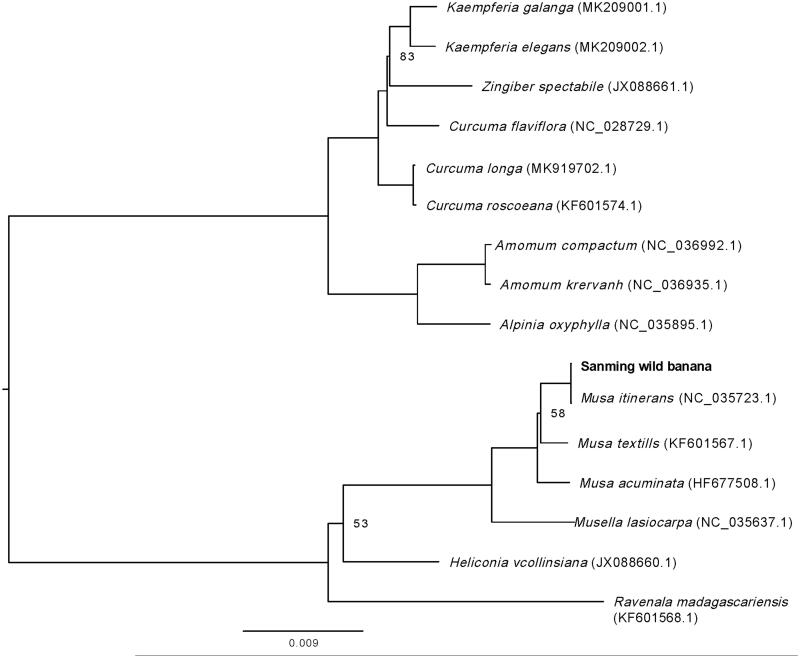
Maximum-likelihood phylogenetic tree based on the complete chloroplast genome sequences of 16 species from the order Scitamineae. Numbers on the nodes are bootstrap values with 1000 replicates and bootstrap values of 100 were omitted.
